# Fibroblast Growth Factor-2 alone as an efficient inducer for differentiation of human bone marrow mesenchymal stem cells into dopaminergic neurons

**DOI:** 10.1186/s12929-014-0083-1

**Published:** 2014-09-24

**Authors:** Sushmita Bose Nandy, Sujata Mohanty, Manisha Singh, Madhuri Behari, Balram Airan

**Affiliations:** Stem Cell Facility, All India Institute of Medical Sciences, New Delhi, India; Department of Neurology, All India Institute of Medical Sciences, New Delhi, India; Department of Cardio Thoracic Vascular Surgery, All India Institute of Medical Sciences, New Delhi, India; Department of Biomedical Sciences, Texas Tech University Health Science Center, 5001 El Paso Drive, El Paso, TX 79905 USA

**Keywords:** hBM MSC, Dopaminergic neurons, FGF2, Shh/FGF8, ATRA, Parkinson’s disease, Calcium ion imaging

## Abstract

**Background:**

The reported efficiency of differentiation of human bone marrow derived Mesenchymal Stem Cells (hBM MSC) into dopaminergic neurons with different inducers is found to vary. Thus, in the current study we have investigated the response of hBM MSC to some of the neuronal inducers and their combinations. Neuronal differentiation inducing agents Fibroblastic Growth Factor 2 (FGF2), Sonic Hedge Hog (Shh), Fibroblastic Growth Factor 8 (FGF8) & All Trans Retinoic Acid (ATRA) were used either singly or in varied combinations.

**Results:**

The differentiated and undifferentiated hBM MSC were characterized in terms of morphology, expression of cell markers at transcriptional and translational levels, amount of dopamine secreted by the cells in the media and changes in cell membrane potential by calcium ions imaging. Induced hBM MSC revealed neuron like morphology and expressed cellular markers suggesting neuronal differentiation with all the inducing agents. However, upon quantitative analysis through qPCR, cells induced with FGF2 were found to show maximum expression of tyrosine hydroxylase (TH) by 47.5 folds. Immunofluorescence analysis of differentiated and undifferentiated cells also revealed expression of nestin, neurofilament, microtubule associated protein- 2, beta tubulin III and TH in differentiated cells, at translational level. This data was supported by immunoblotting analysis. Further, ELISA study also supported the release of dopamine by cultures induced with FGF2. When the cells were depolarised with KCl solution, those induced with Shh & FGF8 showed maximum calcium ion trafficking, followed by the cells induced with FGF2 only.

**Conclusions:**

We conclude that hBM MSC can be coaxed to differentiate efficiently into dopaminergic neurons in the presence of a very simple media cocktail containing only one main inducer like FGF2 and thus contribute towards cellular therapy in Parkinson's and other related disorders. These dopaminergic neurons are also functionally active, as shown by calcium ion trafficking.

**Electronic supplementary material:**

The online version of this article (doi:10.1186/s12929-014-0083-1) contains supplementary material, which is available to authorized users.

## Background

Parkinson’s Disorder (PD) is a neuro-degenerative disease caused by degeneration of nigrostraital system, affecting people worldwide. Progressive degeneration of dopaminergic (DA) neurons in the pars compacta of the substantia nigra is the major cause of the disorder. Stem cells have the potential to generate lineage committed cells, showing great promise to regenerative medicine. Human bone marrow derived Mesenchymal Stem Cells (hBM MSC) are considered as one of the best candidates for regeneration because of their properties like easy isolation, robust expansion, immunological naivety and devoid of ethical issues [1-3]. The stimulation for commitment towards neuronal lineage given to MSC in various studies was in the form of chemicals or growth factors [4-10]. Use of growth factors as inducers of differentiation has led to the generation of better results in terms of efficiency, cytotoxicity, and long term maintenance of these cells in culture, instead of chemicals [11]. The efficiency of tyrosine hydroxylase (TH) positive neuron generation from MSC has been reported variedly. This might be because of the different differentiation protocols followed to provide stimulation to MSC by various research groups. Thus, there is still a need for an optimum protocol which can generate TH positive neurons from hBM MSC.

The focus of the present study is on inducers like FGF2, Shh, FGF8 and ATRA as they all have role in the early developmental stages of the nervous system. Although these inducers have been studied earlier, but they have never been compared in terms of efficiency of TH positive cells generation using hBM MSC. We have explored the formulation of a defined efficient media by investigating the different growth factors and their combinations, with the aim of robust generation of DA neurons from hBM MSC.

## Methods

The study was approved by the Institution Human Ethics Committee.

### MSC isolation, culture and characterization

Normal human BM was collected from patients of age ranging from 13–58 yrs, undergoing Stem Cell Transplantation after taking prior informed consent from patient or legal guardian. Five BM samples, aspirated from iliac crest of the patients have been used in our study. Primary culture of hBM MSC was established and maintained as per described in our previous paper [12]. For all the subsequent experiments, cells from 3^rd^ passage were used.

### Characterization of expanded hBM MSC: flow cytometric analysis

#### Surface marker staining

Surface marker staining to characterize the isolated hBM MSC was done as per the protocol described in our previous publication [12]. CD73 PE, CD90 PECy5, HLA Class I APC, HLA Class II FITC, CD 34 PE (Becton Dickinson, USA) & CD105 APC (e Bioscience, USA) were used. Controls were made by corresponding isotypes: IgG1 coupled with PE, PECy5, APC & FITC.

#### Intracellular staining

hBM MSC after exposure to the differentiation inducing agents, were intracellularly stained for TH and Neuronal Nuclei (NeuN), as per described in our previous protocol [12].

### Cell differentiation: osteogenic, adipogenic, chondrogenic and neuronal lineages

hBM MSC were characterized by differentiating cells into osteogenic, chondrogenic and adipogenic lineages according to the induction protocols described in our earlier published research article [12].

Neuronal Differentiation: Basic induction medium containing Neurobasal medium (Gibco, USA), B27 supplement (Gibco, USA), EGF (Peprotech Asia, Israel), L-Glutamine (Gibco, USA) and PenStrep (Gibco, USA) was used. FGF2 (10 ng/ml), Shh + FGF8 (10 ng/ml), ATRA (0.1µM) and Shh + FGF8 + ATRA were added to the basic induction media in different combinations (All the growth factors were procured from Peprotech Asia, Israel and ATRA from Sigma, USA). The induction protocol was carried out for 12 days in all the four study groups.

### Cell growth kinetics assay

MTT (3-[4, 5-dimethylthiazol-2-yl]-2,5- diphenyltertra-zolium bromide) assay was used to investigate cytotoxic effects of various inducers on hBM MSC. The assay was performed at various time points of induction period (Day1, day3, day5, day7, day9 and day12) in all the four study groups with different inducers. Briefly, 1 × 10^4^ hBM MSC per well were plated onto flat-bottomed 48-well plates (Corning Glass Works, Corning, New York) with 500 μl of induction medium. At termination of different time points, the cells were subjected to 50 μl of MTT solution (5 mg/ml) for three hours at 37°C. Finally, 300 μl dimethylsulfoxide (Sigma, USA) was added to each well and incubated for 30 minutes at 37°C to dissolve all the formazan crystals. The coloured solution (200 µl) was transferred to 96 well plates and read at 570 nm using EL 800 plate reader (Biotek, USA) and recorded with Gen5 1.08.4 software (Biotek, USA). All the experiments were performed in triplicates for three samples each.

### Reverse transcriptase polymerase chain reaction

Total RNA was isolated after termination of the differentiation induction period by phenol-chloroform method. cDNA was prepared using 1 μg of RNA by RT enzyme (Promega, USA). This cDNA was further used for amplification by PCR using primers for neuronal related genes (Table 1). Finally, PCR products were run on 2% agarose gel using gel electrophoresis system (Biorad Laboratories) and analysed on gel documentation system (Alpha Digi Doc).

**Table 1:** **List of primer pairs used for RT-PCR/ qPCR**

**Gene**	**Primer sequence**	**Annealing temp.**
**Nestin**	GCCCTGACCACTCCAGTTTA	55.0°C
	GGAGTCCTGGATTTCCTTCC	
**Neurofilament**	TGGGAAATGGCTCGTCATTT	56.7°C
	CTTCATGGAAGCGGCCACTT	
**TUJ I**	GGGATCCACTCCACGAAGTA	61.0°C
	CGAGACCTACTGCATCGACA	
**MAP2**	CTCAACAGTTCTATCTCTTCTTCA	60.0°C
	TCTTCTTGTTTAAATCCTAACACT	
**TH (For RT & qPCR)**	GGTCGCGCTGCCTGTACT	53.9°C
	TCATCACCTGGTCACCAAGTT	
**GAPDH**	GACAAGCTTCCCGTTCTCAG	57.0°C
	GAGTCAACGGATTTGGTCGTCGT	

### Quantitative PCR (qPCR)

The qPCR experiments were performed using a Realplex real time PCR detection system (Eppendorf, Germany). Human specific primers (MWG-Biotech AG) used are as mentioned in Table I. Reactions were carried out using SYBR Green Super Mix (Kapa Biosystems, USA) in a final volume of 10 μl with 0.3 μM of each primer. Negative controls were used as no template cDNA reactions and melting curves were used to confirm the results. The results were normalized using GAPDH concentration of each sample.

### Immunoflorescence assay

The assay was performed as described previously [12], using primary monoclonal antibodies against nestin, neuronal nuclei (NeuN), microtubule associated protein 2 (MAP2), neurofilament (NF) and β- tubulin III (Tuj1) (Chemicon, USA), followed by incubation with respective secondary antibodies (TR conjugated for TH and FITC conjugated for nestin, NF, NeuN, Tuj1 and MAP2).

### Dopamine estimation through enzyme linked immunosorbent assay (ELISA)

DA levels were quantified using an ELISA kit obtained from Rocky Mountain Diagnostics (Colarado Springs, CO) according to the manufacturer’s instructions. At the end of termination period, media was collected and 4 mM of Sodium Metabisulphite and 1 mM of EDTA was added and stored at −80°C till processed.

### Functional assay for differentiated cells

#### Cell preparation and loading of dye

Human BM MSC were cultured in 2-chambered culture slides (Nunc, Denmark). These cells were exposed to neuronal differentiation media for induction as mentioned above. Post differentiation, these cells were washed twice with 1X HBSS (Sigma, USA) at room temperature under sterile condition. 10 nM of Fura-Red-AM (Invitrogen, USA) solution was made in phenol red free 1X HBSS which was then probed on to the cells in the chambered slides for 45 min at 37°C/5% CO_2_. The dye solution was then removed from the slides and cells were finally washed twice with 1X HBSS to remove the excess non diffused dye. The cells were kept in 1X HBSS for 30 min at 37°C/5% CO2 before acquisition. This was performed for both differentiated cells as well as uninduced (UI) hBM MSC.

#### Visualization and monitoring calcium concentration changes

The experiment was performed using Leica Confocal Microscope (Model TCS SP5). Recording for both the wavelengths (488 nm and 457 nm) were carried out for 6 min in HBSS. Then, HBSS was removed and 50 mM KCl solution was added before further recording for 10 min. Experiments were carried out in duplicates. The data was analysed using Leica LAS AF software. The intracellular calcium concentration was calculated according to the formulae derived by Grynkiewicz et. al. [13].

### Immunoblotting analysis

Whole cell lysates were obtained by resuspension of cell pellets in 500 μl RIPA buffer (Sigma, USA) and 40 μl of protease inhibitor cocktail (Sigma, USA). The lysates were estimated for protein concentration using BCA Assay method. Protein extracts (40 μg) were subjected to SDS-PAGE using 12% Tris/HCl SDS gels and transferred onto polyvinylidene difluoride (PVDF) membranes (Membrane Technologies, India). Membranes were blocked using 3% BSA (Himedia, India) for 2 hours, followed by incubation with primary antibody against β- actin (Abcam,UK, 1:500) and tyrosine hydroxylase (Santa Cruz, USA, 1:500) in 1% BSA- phosphate saline buffer (PBS) overnight at 4°C. Post incubation, membranes were washed thrice in TBST and incubated with the appropriate horseradish peroxidase (HRP) - conjugated secondary antibody (1/4,000) (Dako, USA) for 2 hours at room temperature. Membranes were developed with chemiluminescence detection reagents (Pierce, USA) and acquired by using Gel Imager machine (Fluor Chem E, Cell Biosciences, Australia).

### Statistical analysis

Means ± SD of independent experiments were analyzed by One-way ANOVA test. P < 0.05 was considered statistically significant.

## Results

### Characterization of human hBM MSC

**Figure 1:**
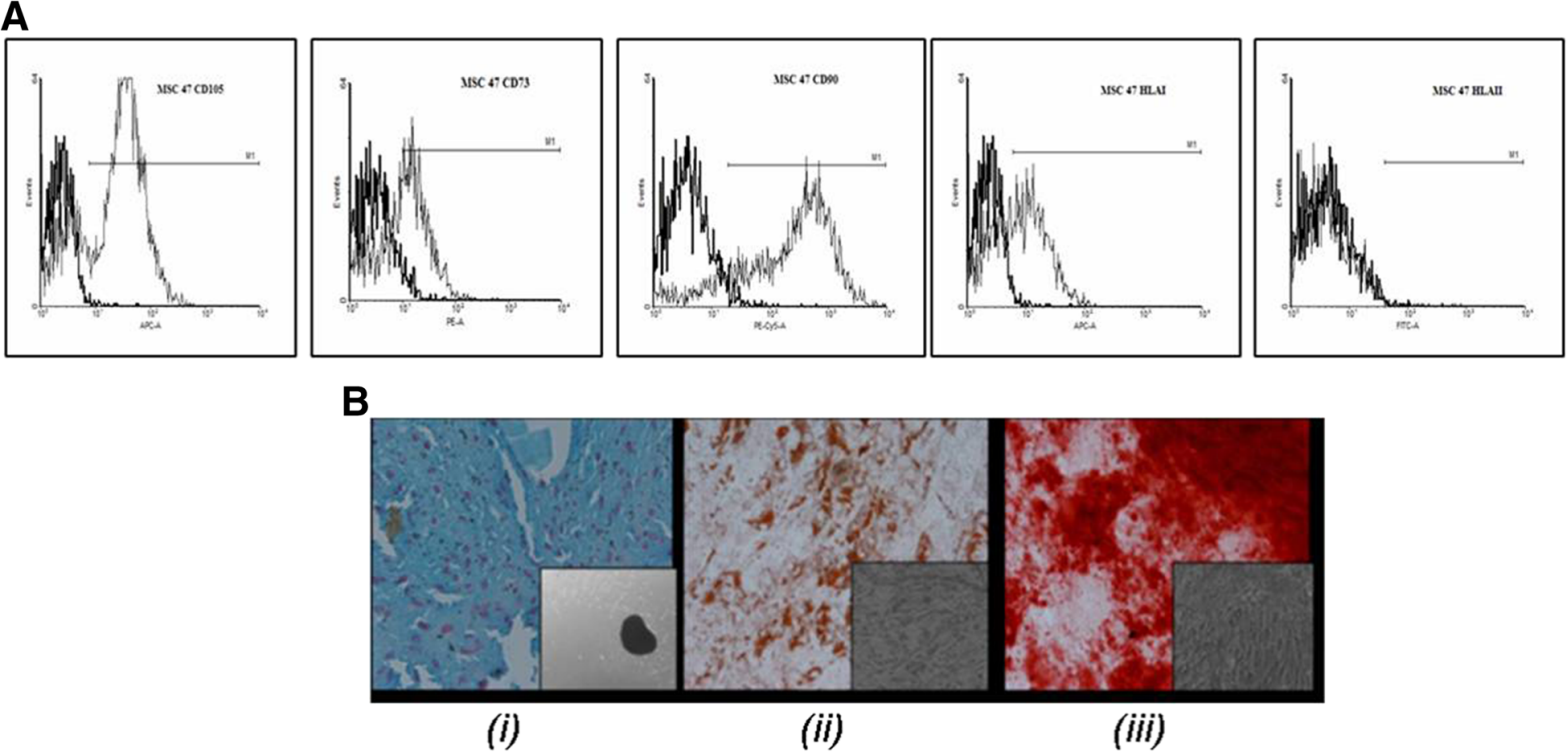
**Characterization of hBM MSC: (A) Flow Cytometric surface marker expression profile (B) Trilineage differentiation capacity: (i) Alcian Blue (ii) Oil Red O and (iii) Alizarin Red S positive staining indicates differentiation towards Chondrogenic, Adipogenic and Osteogenic lineage, respectively.**

The hBM MSC were characterized on the basis of immunophenotyping and tri-lineage differentiation assay. They expressed CD105, CD73, CD90 and HLA Class I and were negative for HLA Class II, CD45 and CD34 (Figure 1A and Additional file 1: Figure S1). These cells were able to appreciably differentiate into adipocytes, osteocytes and chondrocytes after induction (Figure 1B)

### Different differentiation protocols lead hBM MSC towards neuronal lineage

#### Morphological analysis of induced hBM MSC

**Figure 2:**
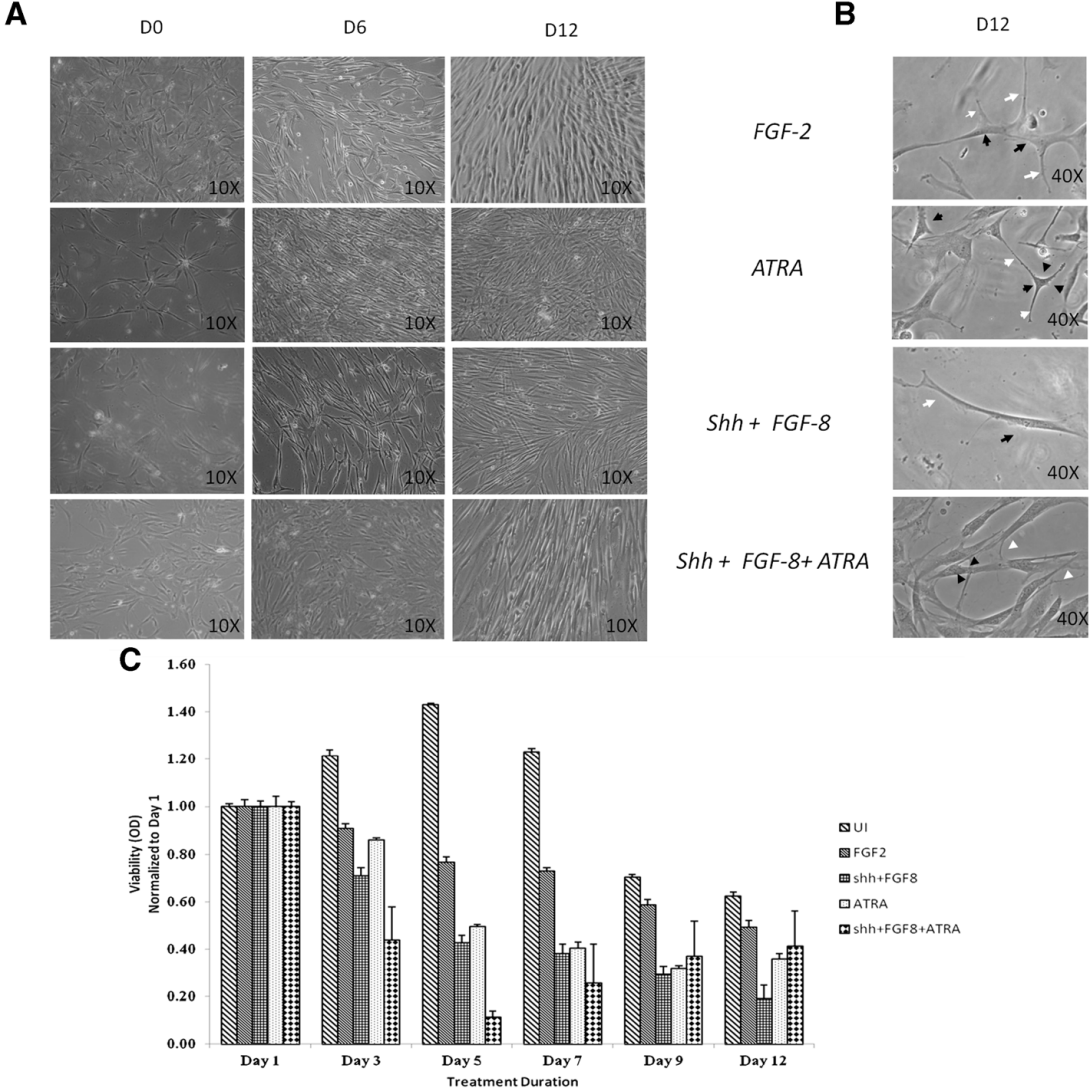
**Morphological examination of hBM MSC neuronal differentiation by bright field microscopy (Day 0, Day 6 &Day 12) and MTT Assay: (A) Cells cultured under different media conditions appeared to have attained neuronal like morphology by Day 6. (B)** Observations under higher magnification (40X) revealed the appearance of extended long cellular processes (white arrows) and retracted cell bodies (black arrow). **(C)** MTT Assay of uninduced (UI) and induced hBM MSC at Day3, Day5, Day7, Day9 and Day12;normalized with day1.

Analysis of morphological features revealed the transition from typical hBM MSC morphology i.e. spindle shaped with monolayer arrangement (whirlpool pattern) towards neuronal like cells during the 12 day induction period (Figure 2 A-B). Maximum cell death was found in media with Shh + FGF8 + ATRA followed by Shh + FGF8, ATRA and least in media with FGF2, as depicted by the MTT assay at various time points of neuronal induction (Figure 2C).

#### Expression of neuronal specific markers in hBM MSC

**Figure 3:**
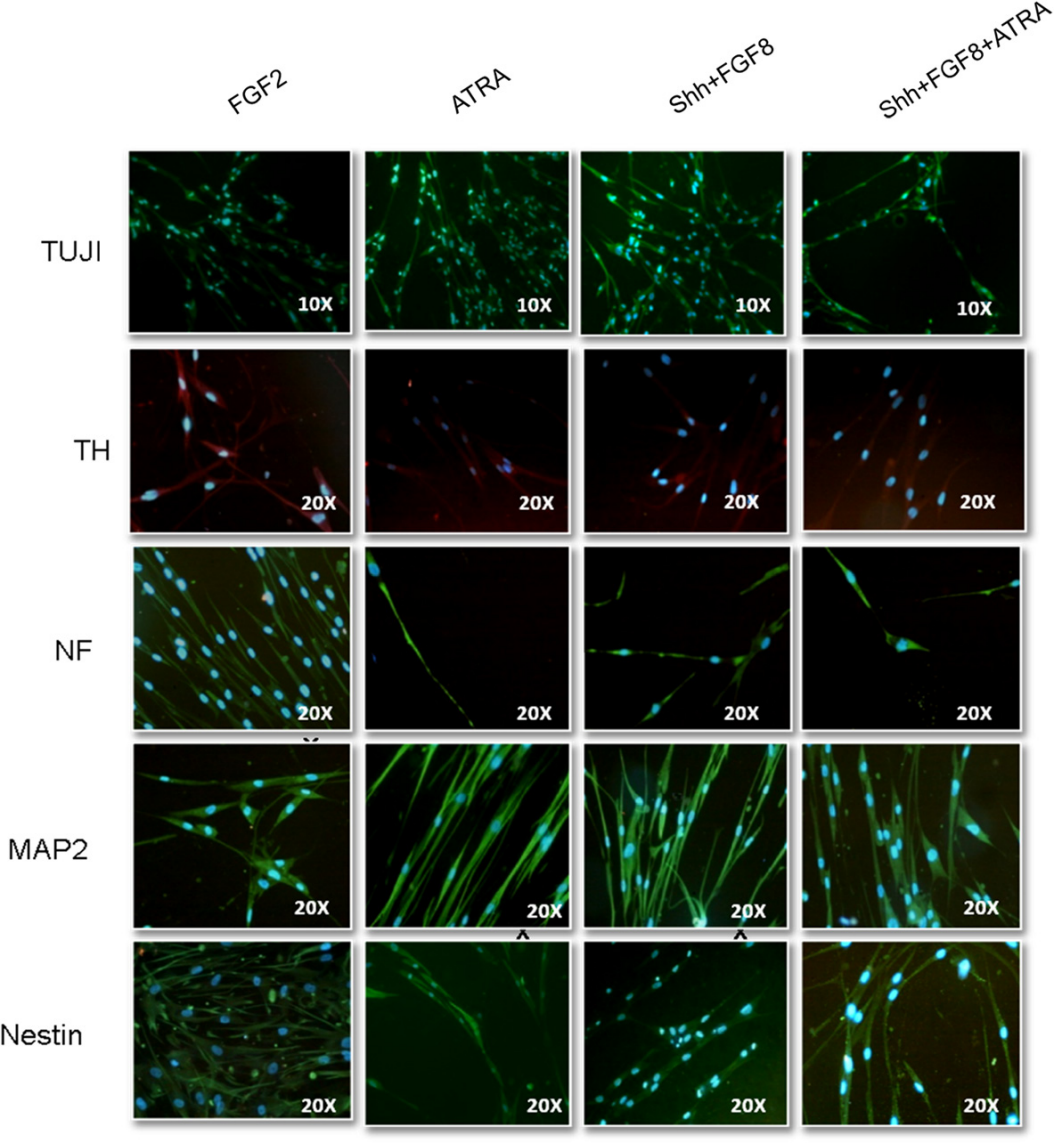
**Expression of neuronal specific proteins after 12 days of induction: cells cultured with different induction media showed positivity for TUJI, TH, Neurofilament (NF), MAP2 and Nestin.** Tyrosine Hydroxylase (TH) was found to be dimly positive in cells treated with various induction media.

**Figure 4:**
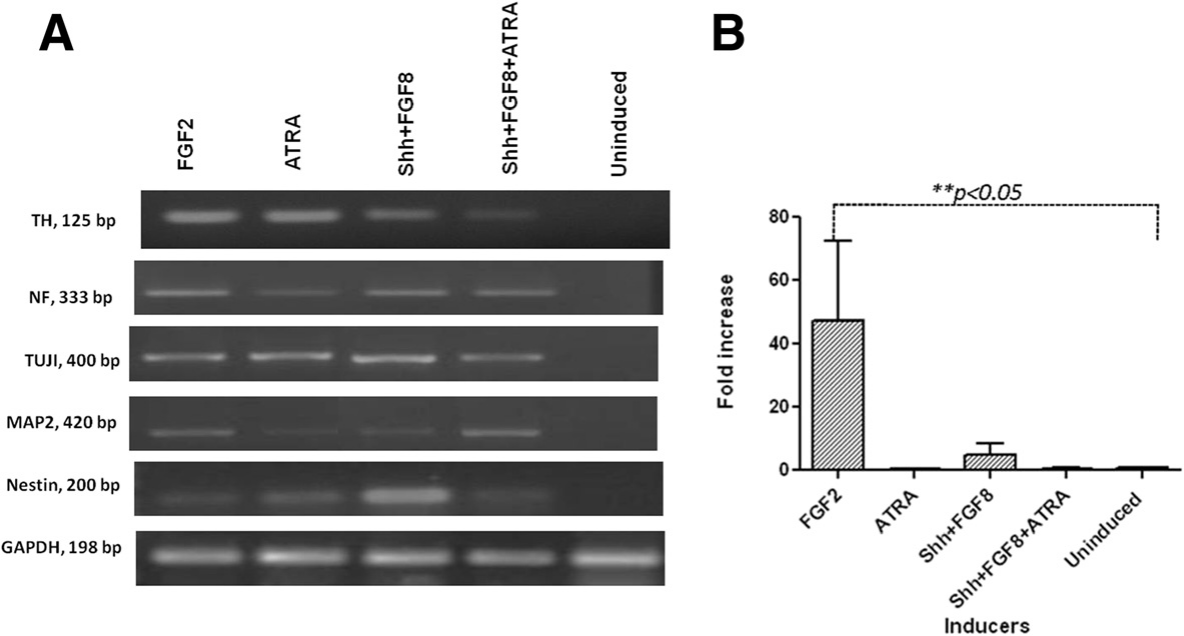
**Molecular characterization for neuronal lineage related genes: A) Gel electrophoresis image showing the gene expression of TH, Neurofilament, TUJI, MAP2 and Nestin.** GAPDH was used as the internal control. **B)** qPCR graph indicating the fold increase in the expression of TH as compared to its expression in uninduced cells. GAPDH was used for normalization. Cells treated with only FGF2 showed 47.5 folds increase in expression of TH (*p < 0.05).

**Figure 5:**
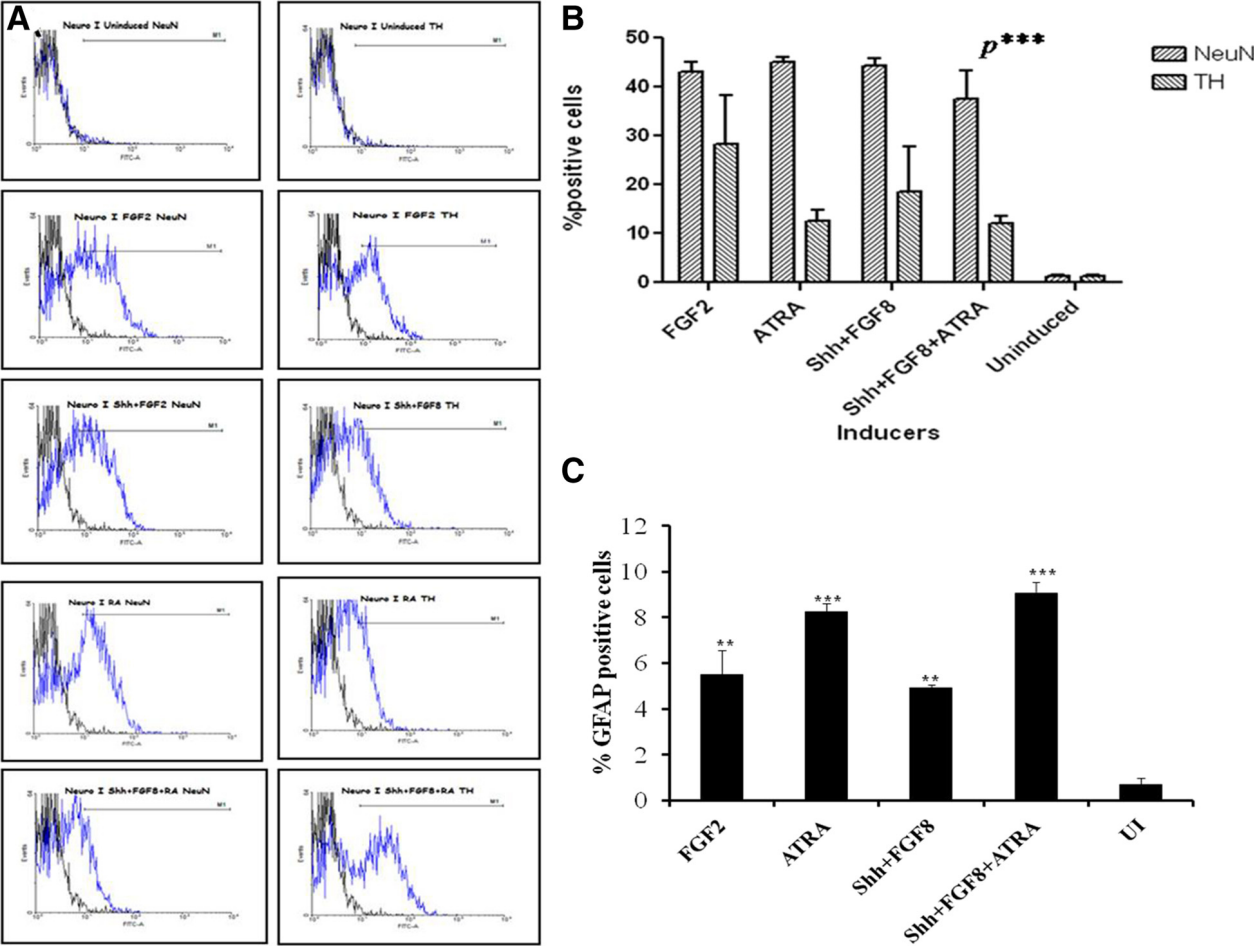
**Flow cytometric analysis of neuronal markers. A)** Representative plots showing the expression of NeuN and TH in cells obtained from a single culture. **B)** Graph showing the% positive NeuN and TH cells in a single culture for all the induction media. **C)** Graph showing the% positive GFAP cells in all the induction media.

The different media were able to coax hBM MSC towards neuronal lineage as this was evident by the observations of transcriptional and translational studies. The expression of Nestin, NF, TUJI, MAP2 and TH were checked through immunofluorescence (Figure 3) and Reverse Transcriptase-PCR (Figure 4A) analysis. The results revealed the expression of all these markers, indicative of neuronal cell identity. Quantitative Flow cytometric analysis for NeuN expression in different induction protocols revealed equivalent prevalence of this marker for all cultures treated with different media (Figure 5B).

### Induced hBM MSC showed traits of DA neurons

**Figure 6:**
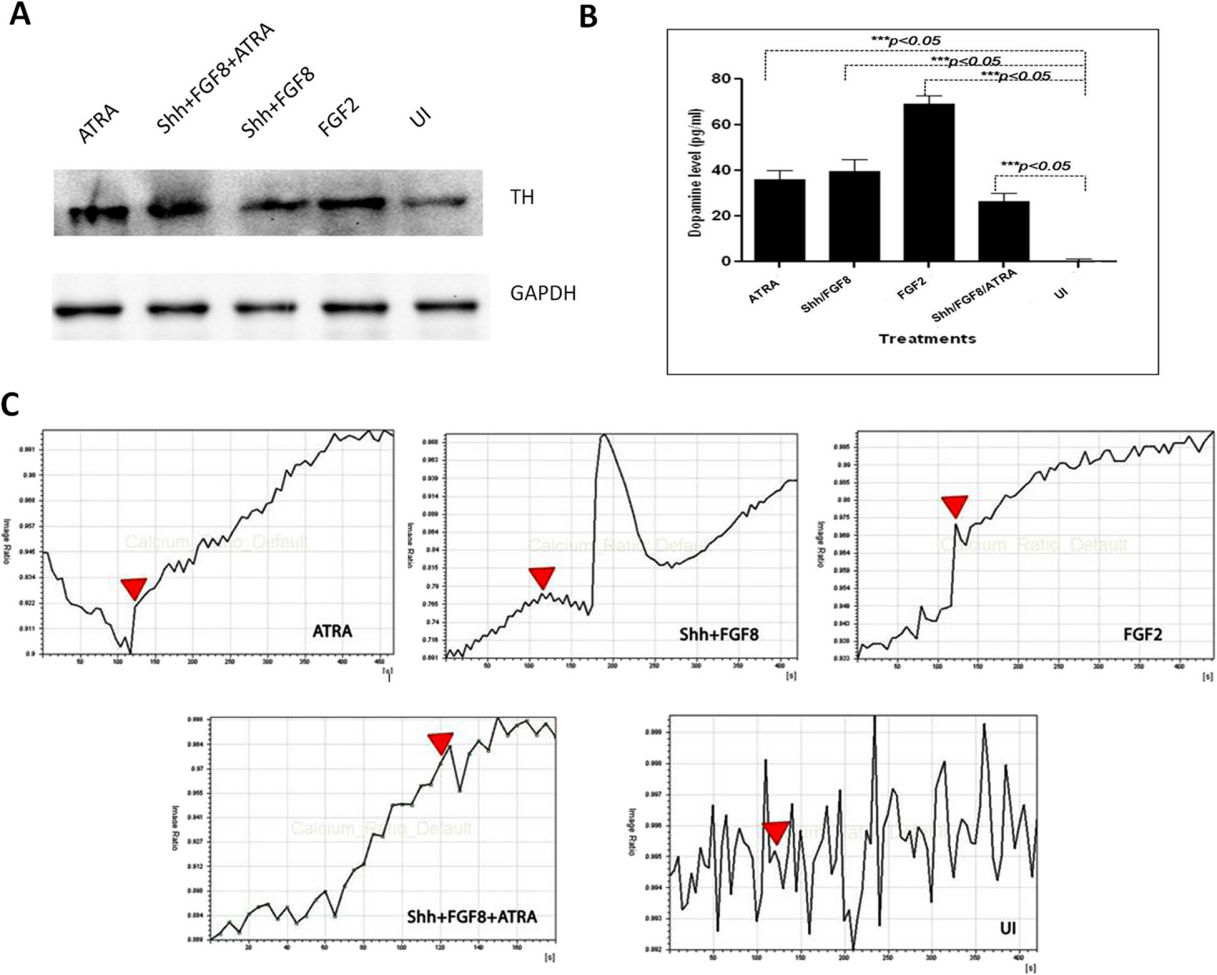
**Dopamine release and Ca2+ imaging analysis of MSC derived DA neurons induced with FGF2, Shh/FGF8, ATRA, and Shh/FGF8/ATRA A) Western Blot showing expression of TH in hBM MSC induced with different media cocktails and a baseline in UI group. B)** Graph showing the level of dopamine released in the media by hBM MSC upon induction with different inducers under study. Maximum concentration of dopamine was released by cells induced with FGF2 only as neuronal inducer. **C)** Graphs represent treatment with FGF2 induces elevated Ca2+ transient upon depolarization with KCl along with other culture media. The uninduced MSC doesn’t show any net change in the intracellular Ca2+ concentration after depolarization. The block arrows indicate the addition of KCl for depolarization.

Presence of TH was observed at both translational and transcriptional levels. Immunofluorescence analysis revealed the presence of TH positive neurons in all the differentiation protocols (Figure 3 & 4A-B). Quantitatively, through flow cytometry, TH was found to express in 28.18 ± 10%, 12.5 ± 2.3%, 18.6 ± 9.1% and 12.06 ± 1.5% of cells in media with FGF2, ATRA, Shh + FGF8 & Shh + FGF8 + ATRA respectively, against almost equivalent percentage expression of NeuN in cell cultures treated with different media (Figure 5A-B). Basal level of expression of TH and NeuN was 1.25 ± 0.2% and 1.27 ± 0.23% respectively. Cells treated with media containing FGF2 showed maximum up regulation of TH followed by Shh/FGF8, Shh/FGF8/ATRA and ATRA (Figure 4B) when evaluated through quantitative PCR. Quantitative PCR data was also supported by western blot analysis. However, lower level of TH was observed in the uninduced group (Figure 6A).

### Post induction culture is heterogenous: presence of astrocytic population

Flow cytometric analysis revealed that 9% of induced culture populations were positive for GFAP in case of media with Shh + FGF8 + ATRA followed by media containing ATRA (8.3%), FGF2 (5.5%) and Shh + FGF8 (4.9%) (Figure 5C).

### TH positive neurons release dopamine in culture supernatant

The DA levels were measured with an ELISA kit based on the principal of competitive ELISA. Neurobasal Media and Neurobasal media + B-27 supplement were used as controls. Dopamine was detected only in cells treated with media containing FGF2, at a concentration of 69.1 ± 3.9 pg/ ml (Figure 6B)

### Differentiated cells demonstrate active intracellular calcium changes in response to potassium chloride (KCl)

It is known that functional neurons express calcium channels which get activated by depolarization. We observed that stimulation with depolarizing agent, KCl; led to an increased calcium influx in the cells induced for differentiation (Additional file 2: Figure S2). It was presumably due to the activation of calcium channels. Interestingly, we did not observe any net change in the intracellular calcium influx before and after depolarization in UI MSC. Figure 6B shows the representative software generated graph of image ratio for all the treatment groups as well as the UI MSCs which have been supplemented with time lapse calcium imaging videos (See video uploads in Additional files 3, 4, 5, and 6: M1/FGF2, M2/FGF8 + Shh, M3/ATRA, M4/ Shh + FGF8 + ATRA).

## Discussion

hBM MSC pre-differentiated towards DA neurons can contribute to the treatment of PD without raising issues of rejection, malignancy and ethics as in case of Embryonic Stem Cells (ESC). Much efforts have been laid to investigate the appropriate *in vitro* cues which drive these cells to differentiate into TH positive neurons. hBM MSC induction by ATRA [7], Shh [15], FGF8 [17] and FGF2 [11] has been widely used for neuronal differentiation. These induction strategies are based on the *in vivo* role of these factors during the development of the nervous system in embryonic stage. However, considering the previous literature, not much has been elaborated on the *in vitro* effect of these factors on hBM MSC, especially in the process of DA neuron generation. Thus, in this study we tried to elucidate the effect of these exogenous factors on BM MSC and provide with a comparative overview of the same.

The dose of Shh and FGF8 used in the current study is less as compared to that used in other studies involving ESC or MSC, in which Shh and FGF8 have been used in the range of 250–500 ng/ml and 100–250 ng/ml respectively. In our case, during initial standardization we have noticed that the higher concentrations of Shh and FGF8 were cytotoxic to the hBM MSC and the cells showed loss of adherence. We reduced the concentration to 10 ng/ml for both Shh and FGF8 after titration with concentrations 250, 200, 150, 100, 50, 25, 20 and 10 ng/ml. At the lowest concentration of 10 ng/ml of Shh and FGF8, we observed no significant cytotoxicity and maintenance of the adherence properties. One reason for this contradiction may be the use of adherence substrates like laminin and fibronectin by the previous studies.

The time period of induction of DA neurons generation in stem cells has shown variation ranging from 3 to 21 days. Especially in case of ESCs and sequential directed differentiation, the induction period is long as compared to the case of hBM MSC, where majority of studies have reported an induction period of not more than 2 weeks. In our study also, it was observed that the cells beyond 2 weeks were not healthy and there was increase in cell death. Thus, we optimized the induction period to day 12, after which, upon characterization we found the expression of neuronal markers as well as traits of DA neurons.

With the development of techniques and protocols to generate DA neurons, different types of strategies have been investigated, amongst which, sequential directed differentiation has been extremely common; with two variations, one with the use of chemical reagents and another with cytokines/ growth factors. In majority of cases, cells have been pre-primed with FGF2 before the initiation of the induction process. However, there was no report about the status of the DA neuron related molecular markers after treatment with FGF2 in these cells.

Upon getting positive cues in the direction of DA neuron generation, during the initial experiments using hBM MSC, we planned to include FGF2 as one of the inducers in the current comparative study. Also, FGF2 is reported to be involved in the development, maintenance, & survival of the nervous system [14]. It exerts neurotrophic activity on DA neurons both *in vitro* and *in vivo*. FGF2 along with Epidermal growth factor (EGF) and Platelet derived growth factor (PDGF) has previously been used for *in vitro* differentiation of MSC into neurons [11]. Apart from this, many other studies with the aim of DA neuron differentiation have used FGF2 for pre-priming of stem cells before exposure to the specific induction agents. However, its role in terms of efficient DA neuron generation needs to be evaluated in hBM MSC.

ATRA, through the Retinoid signaling pathway acts as an important mediator of cell differentiation and function during the development of the nervous system [15]. ATRA mediated *in vitro* induction of hBM MSC has shown differentiation towards neuronal lineage as analysed by the presence of neuronal specific markers. In the present study, ATRA alone and in combination with Shh and FGF8 was used, as these factors have shown to exert synergistic effects on neural competent MSCs to induce expression of a comprehensive set of genes and proteins that define peripheral nervous system (PNS) sensory neurons [16].

Shh and FGF8 have been specifically used in the differentiation of ESC into TH positive neurons, *in vitro*. It is believed that developing cells *in vivo* are signaled to differentiate into DA phenotype when they encounter intersecting signals occurring along the anterior-posterior (FGF8) and dorsal-ventral (Shh) axes [17]. In the present study, combination of both Shh and FGF8 is used as it has been observed previously that neither of the GFs i.e. Shh and FGF8 alone can adequately provide signal for DA neuron generation *in vivo* and the presence of both Shh and FGF8 simultaneously lead to the appearance of TH positive DA neurons

The present study comparatively evaluates the optimal inducer of neuronal lineage with significant inclination towards generation of neuronal subtype i.e. DA neurons from hBM MSC. As of now, very few studies have compared the chemicals/GFs to determine optimum induction media composition [18,19]. Different studies with the use of varied induction protocols reported induction efficiency of MSCs to DA neurons ranging from 12.7 to 67% [9,20-23]. Katarzyna *et al.,* 2007 have reported the presence of 90% neurons as depicted by expression of the neuronal mature marker NeuN in differentiated hBM MSC when induced with Shh + FGF8 + FGF2 with an efficiency of ~ 67% to generate TH positive neurons (23). However, this and many other such studies do not throw light on the role of FGF2 alone in the differentiation of hBM MSC into dopamine secreting neuronal cells. Our study reveals a maximum efficiency of 66% with FGF2 alone as induction agent followed by Shh + FGF8 (42%), Shh + FGF8 + ATRA (32%) and ATRA (27.6%). This suggests that the DA neuron induction efficiency with only FGF2 is comparable in terms of induction efficiency obtained with Shh + FGF8 + FGF2. If we compare the induction studies of Katarzyna *et al. (23)* and ours, culture media containing FGF2 along with Shh and FGF8 fairs better than FGF2 alone as used in our study in terms of overall percentage of neuronal differentiation.

This study also indicated the presence of glial fibroblast acidic protein (GFAP) positive cell population in the induced cultures using different differentiation protocols. This can be a promoting and supporting factor for the generation and survival of DA neurons. It has also been reported that *in vivo* astrocytes are capable of regulating neurogenesis by apparently instructing the stem cells to adapt a neuronal fate [24]. A majority of previous studies have used glial derived neurotrophic protein (GDNF) or brain- derived neurotrophic factor (BDNF) as one of the constituents of the differentiation media cocktail [21]. However, it seems that the GFAP positive cells don’t have any role in neuronal sub type determination during *in vitro* differentiation.

We further investigated the functionality of these differentiated cells. Calcium ion acts as an important secondary messenger for signal transduction. Intracellular Ca^2+^ concentration changes caused by extracellular high K^+^ ions in cells is usually used as an indicator of excitability [25]. At variance with previous reports regarding hBM MSC [26,27], we and other groups have detected these features only after neuronal differentiation, but not in uninduced state. In the current study, we have found that there was a certain level of calcium activity post depolarization with KCl in neuronal differentiated MSCs. Cells treated with different induction media consisting of FGF2, Shh/FGF8, ATRA and Shh/FGF8/ATRA showed change in Ca^2+^ concentration upon depolarization. A total change of 8 folds in the calcium ion concentration was observed in case of media containing FGF8 and Shh, followed by 6.5 fold change in media with FGF2 alone. Although, the change in the calcium ion concentration with remaining two groups of media cocktails was found to be insignificant.

The present results are in line with previous reports stating the role of FGF2 and Shh + FGF8 during DA neuronal differentiation *in vitro* as well as *in vivo*. These were the two induction agents which yielded maximum DA neurons differentiated from hBM MSC. We conclude that FGF2 alone is an adequate signal to drive hBM MSC towards DA neuronal subtype.

## Conclusion

FGF2 alone proves to be an efficient inducer to differentiate BM-MSC into functionally active dopaminergic neurons.

## Additional files

Additional file 1: Figure S1. Graph showing expression of surface markers for characterization of hBM MSC by Flow Cytometry. Additional file 2: Figure S2. Change in concentration of calcium ion efflux by cells induced with different inducers pre- and post- KCl stimulation. Additional file 3
**The video recordings for pre-and post- KCl stimulated cells from all induced and control groups.** The change in fluorescence upon KCl stimulation is indicative of calcium efflux. They represent calcium efflux analysis for hBM MSC induced with FGF2. Additional file 4
**Shh+FGF8.**
Additional file 5
**ATRA.**
Additional file 6
**Shh+FGF8+ATRA.**

## Abbreviations

hBM MSC: human Bone Marrow Mesenchymal Stem Cells
BM MSCs: Bone marrow- mesenchymal stem cells
FGF2: Fibroblast growth factor-2
SHH: Sonic hedgehog
FGF-8: Fibroblast growth factor-8
ATRA: All- trans retinoic acid
PD: Parkinson’s disease
DA: Dopaminergic
TH: Tyrosine hydroxylase
NeuN: Neuronal nuclei
NF: Neurofilament
MAP-2: Microtubule associated protein-2
Tuj1: β- tubulin III
ELISA: Enzyme linked immunosorbent assay
ESC: Embryonic stem cells
EGF: Epidermal growth factor
GFAP: Glial fibrillary acidic protein
