# Supplementary figures and images for: Fibroblast Growth Factor-2 alone as an efficient inducer for differentiation of human bone marrow mesenchymal stem cells into dopaminergic neurons
Source: J Biomed Sci. 2014 Sep 24;21(1):83. doi: 10.1186/s12929-014-0083-1 (PMC4190371; doi:10.1186/s12929-014-0083-1)

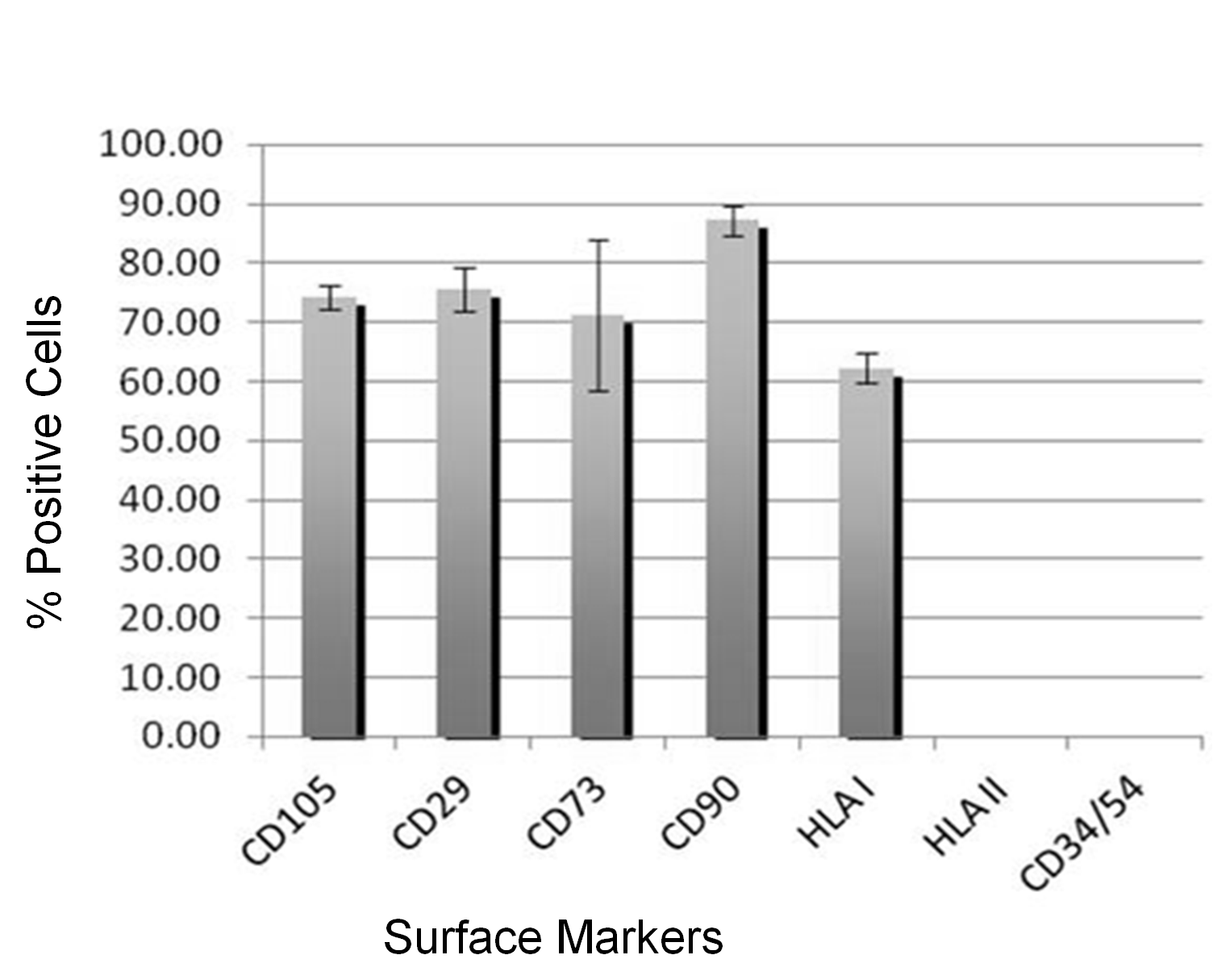

Supplement: Additional file 1: Figure S1. — Graph showing expression of surface markers for characterization of hBM MSC by Flow Cytometry. [file 12929_2014_83_MOESM1_ESM.tiff]

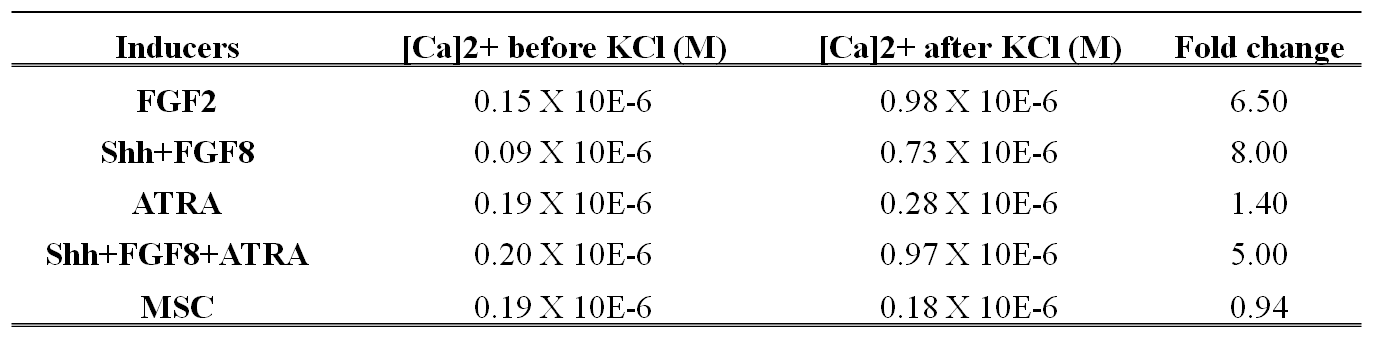

Supplement: Additional file 2: Figure S2. — Change in concentration of calcium ion efflux by cells induced with different inducers pre- and post- KCl stimulation. [file 12929_2014_83_MOESM2_ESM.tif]
